# Supplementary material for: Defective Integrator activity shapes the transcriptome of patients with multiple sclerosis
Source: Life Sci Alliance. 2024 Jul 19;7(10):e202402586. doi: 10.26508/lsa.202402586 (PMC11259605; doi:10.26508/lsa.202402586)
Supplement: Supplementary file 4 [file LSA-2024-02586_TableS4.docx]

**Sup. Table 4: RT-qPCR primers**

| **Name** | **Sequence** | **Amplicon (bp)** |
| --- | --- | --- |
|  |  |  |
| CBX5-e3-F | AACAGTGCCGATGACATCAAA | 110 |
| CBX5-e4-R | GCCCCAATGATCTTTTCTGGT |  |
|  |  |  |
| INTS6-e5-F | CTGGCACCATGTCAGTAGAAT | 116 |
| INTS6-e6-R | TCTTGGAGAACACACAGAATATGA |  |
|  |  |  |
| INTS8-e12-F | GTTGATCAGATGAGGAAGAGATCC | 98 |
| INTS8-e13-R | CCAATGTTGACACTTGCTGAAG |  |
|  |  |  |
| INTS3-e20-F | CTAGACCTTCTCTCCGAGCTAT | 110 |
| INTS3-e21-R | GACTCGTACAGGTTCATCTTCC |  |
|  |  |  |
| HPRT-e2-F | TATGGACAGGACTGAACGTCTTGC | 80 |
| HPRT-e3-R | TGAGCACACAGAGGGCTACAAT |  |
|  |  |  |
| RPLP0-e6-F | AGGTGTTCGACAATGGCAGCAT | 112 |
| RPLP0-e7-R | TGCAGACAGACACTGGCAACAT |  |
|  |  |  |
| IRF2-in1-F | TGTGTATGCGTGTCCTGATG | 106 |
| IRF2-in1-R | CATAATTGCGCCGACTGTTC |  |
|  |  |  |
| IRF2-in7-F | CCACATCCCGTGATCTCATT | 119 |
| IRF2-in7-R | GCAAAGTGGCTGAGTGTTTC |  |
|  |  |  |
| AHI1-in1-F | CCTTTCCTACTCCCGATTCTTATT | 113 |
| AHI1-in1-R | AGGCTTTGACAGGTTGAAGAT |  |
|  |  |  |
| AHI1-in27-F | AGTGGTCAGAGAAAGCCAAAG | 91 |
| AHI1-in27-R | TCCTGACTTCTAGGCCCTATTC |  |
|  |  |  |
| MBP-in1-F | CATTTGCCGCCACACTAGAT | 126 |
| MBP-in1-R | CCCAAAGACGCTTGAACCTT |  |
|  |  |  |
| MBP-in3-F | GCCAAGCTACACATTCAGAGA | 104 |
| MBP-in3-R | GATGTGGTAGAAGGGCAATAGG |  |
